# Choriocapillaris Flow and Retinal Vascular Fractal Dimension in Dry Age-Related Macular Degeneration †

**DOI:** 10.3390/diagnostics16030422

**Published:** 2026-02-01

**Authors:** Mine Ozturk, Abdullah Ağın

**Affiliations:** Department of Ophthalmology, University of Health Sciences, Haseki Training and Research Hospital, 34098 Istanbul, Türkiye; dr_mine@yahoo.com

**Keywords:** age-related macular degeneration, fractal dimension, choriocapillaris flow

## Abstract

**Background/Objective:** To evaluate the association between optical coherence tomography angiography (OCTA)-derived choriocapillaris flow (CCflow), retinal vascular fractal dimension (FD), and drusen burden in eyes with dry age-related macular degeneration (AMD). **Methods:** This retrospective study included 113 eyes from 73 patients with dry AMD. Eyes were classified into large and small drusen groups based on median drusen area. OCTA-derived CCflow and FD indices of the superficial and deep capillary plexuses were analyzed. Patient-level clustered analyses were performed using linear mixed-effects and generalized estimating equation models to account for inter-eye correlation. **Results:** Eyes with large drusen showed significantly lower CCflow compared with those with small drusen (*p* < 0.001), whereas FDsup did not differ between groups, and FDdeep demonstrated only a near-significant trend toward higher values. CCflow was moderately and negatively correlated with drusen area (ρ = −0.452, *p* < 0.001), whereas FDdeep showed no significant correlation in unadjusted analyses (ρ = 0.137, *p* = 0.148). In patient-level age-adjusted multivariable models accounting for inter-eye dependency, CCflow remained independently associated with drusen burden, while FDdeep demonstrated an independent association only after adjustment for age. **Conclusions:** Reduced CCflow is independently associated with increased drusen burden in dry AMD. FD metrics provide complementary descriptive information regarding microvascular remodeling but do not function as independent biomarkers. CCflow may serve as a robust quantitative indicator of early choroidal compromise in dry AMD.

## 1. Introduction

Age-related macular degeneration (AMD) is one of the leading causes of visual impairment and blindness among older adults worldwide, particularly in developed countries [1,2,3,4,5]. As a progressive, degenerative disease affecting the macula, the central region of the retina responsible for sharp vision, AMD is characterized by its two primary forms: dry (atrophic) and wet (neovascular or exudative). While the dry form accounts for the majority of AMD cases, with approximately 85% of patients affected, the wet form is responsible for the most severe vision loss due to abnormal blood vessel growth under the retina, leading to bleeding and scarring [6,7]. The multifactorial nature of AMD, which involves genetic, environmental, and metabolic factors, continues to be a focus of extensive research [8,9,10,11].

Understanding and quantifying the progression of AMD, particularly in its dry form, remain a significant challenge. Although the progression of dry AMD is generally slower compared to the wet form, it is irreversible. It ultimately leads to central vision loss as retinal pigment epithelium (RPE) cells degenerate and photoreceptor layers atrophy. Clinically, several imaging techniques such as fundus fluorescein angiography (FFA), Optical Coherence Tomography (OCT), and OCT Angiography (OCTA) have been developed to monitor the structural and vascular changes in the retina and choroid, which are associated with the progression of AMD. Among these techniques, Fractal Dimension (FD) has emerged as a promising tool for non-invasive quantitative assessments of retinal and choroidal structures [12,13,14,15,16].

OCTA is an advanced imaging technology that allows the visualization of retinal and choroidal microvasculature without the need for dye injection, offering significant advantages over conventional angiography methods such as fluorescein angiography [17]. By detecting motion contrast from red blood cells within vessels, OCTA provides detailed maps of both retinal and choroidal blood flow, enabling the evaluation of vascular abnormalities in AMD. In particular, OCTA has been used to detect early changes in the choriocapillaris and to identify areas of reduced perfusion, which are believed to play a role in the progression of AMD [18]. Recent studies have also suggested that changes in the choroidal vasculature, such as choroidal thinning and decreased choroidal blood flow, are associated with AMD pathogenesis, making the study of choroidal parameters a critical component of AMD research [19,20,21].

FD is another mathematical and geometrical tool used to describe the complexity of retinal and choroidal vasculature [12]. In the context of AMD, FD provides a quantitative measure of the branching complexity and density of blood vessels, offering insights into vascular remodeling and ischemia [12]. There is currently no comprehensive data in the literature regarding FD alterations, especially in patients with dry AMD.

In this study, we aim to evaluate the relationship between OCTA and FD in patients with AMD, focusing exclusively on dry AMD patients. Unlike traditional studies that include a healthy control group, our study exclusively focuses on patients with dry AMD without systemic diseases such as diabetes or hypertension. This approach eliminates confounding effects arising from baseline variability and allows for a more accurate assessment of internal correlations between structural parameters and disease severity. By doing so, we hope to provide a better understanding of how these imaging and quantitative metrics can be used to assess disease progression and severity in AMD patients.

Given the multifaceted nature of AMD, which involves not only retinal but also choroidal components, our study will provide a comprehensive assessment of both retinal and choroidal changes using these advanced imaging techniques. The integration of OCTA and FD analyses will allow us to develop a more holistic view of how vascular and structural changes contribute to the progression of AMD, offering new insights into potential biomarkers for disease monitoring and therapeutic targets. This manuscript is an extended and substantially revised version of a preliminary study that was previously presented as an abstract/poster at the EURETINA 2024 Congress (Barcelona, Spain, 19–22 September 2024) [22].

## 2. Patients and Methods

### 2.1. Study Design and Participants

The study included 73 patients (42 females and 31 males). Bilateral involvement was present in 40 patients (80 eyes), while 33 patients contributed a single eye, yielding a total of 113 eyes included in the analysis. Patients with systemic comorbidities such as diabetes or hypertension were excluded. A diagnosis of dry AMD was confirmed through clinical fundus examination and optical coherence tomography (OCT). Patients with wet AMD, diabetic retinopathy, glaucoma, or other retinal pathologies were excluded. Additional exclusion criteria included significant media opacities impairing image quality or a history of ocular surgery within the past six months. This study was approved by the Institutional Review Board and Ethics Committee of Haseki Training and Research Hospital (no 97-2025, approval date 25 June 2025). The methods complied with the principles of the Helsinki Declaration. Informed consent was waived due to the retrospective nature of the study. Eyes were further divided into two groups based on total drusen area as measured on color fundus photography: large drusen (*n* = 56) and small drusen (*n* = 57), using the median split method.

### 2.2. OCTA Imaging and Analysis

**Figure 1:**
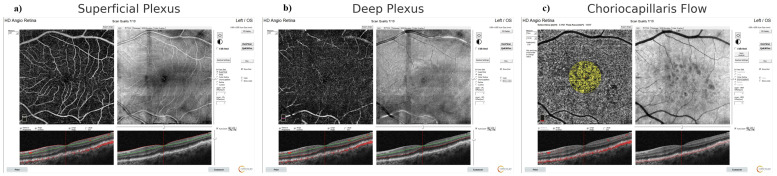
**Representative OCTA Images Used in Analysis.** (**a**) Superficial capillary plexus slab, (**b**) Deep capillary plexus slab, (**c**) Choriocapillaris flow map (yellow overlay). Cross-sectional B-scans below each en face image indicate segmentation accuracy. All images were acquired using the RTVue XR Avanti system (Optovue Inc., Fremont, CA, USA). Scale bar = 1 mm.

After a comprehensive ophthalmologic examination, OCTA (AngioVue, Optovue, Fremont, CA, USA) evaluation was performed. The device automatically calculated parameters, including foveal avascular zone (FAZ), FAZ perimeter, foveal vessel density, subfoveal and peri-parafoveal vessel density (superficial and deep capillary plexus in four quadrants), outer retina, and choriocapillaris flow [outer retina (OR) flow- choriocapillaris (CC) flow]. Segmentation errors were reviewed and manually corrected by trained retinal specialists (MO, AA), blinded to group assignment. Only scans with signal strength ≥ 7/10 and without significant motion artifacts were included in the analysis. Representative en face images demonstrating segmentation of superficial and deep capillary plexuses, as well as choriocapillaris flow, are shown in Figure 1.

### 2.3. Fractal Dimension Analysis

The FD index was used as a quantitative measure of microvascular complexity in the superficial and deep capillary plexuses. OCTA en face images were exported and processed using ImageJ software (v1.54g, National Institutes of Health, Bethesda, MD, USA). Images were first converted to 8-bit grayscale, then standardized and cropped to ensure consistency across samples. Afterward, binarization and skeletonization were applied to isolate the vascular network. The following macro command was applied within ImageJ to generate binary masks:

setAutoThreshold(“Default dark”);

setThreshold(0.0504, 0.1395);

setOption(“BlackBackground”, false);

run(“Convert to Mask”);

The binarized vascular maps were then analyzed using Fractalyse software (v3.0, ThéMA, Besançon, France), which applies the box-counting method to calculate FD. In this approach, the binarized and skeletonized vascular image is overlaid with grids of decreasing box size (r). The fractal dimension is then calculated as the slope of the linear regression of log N(r) versus log (1/r), where N(r) denotes the number of boxes containing vessel pixels at each scale, reflecting the geometric complexity of the vascular network.

**Figure 2:**
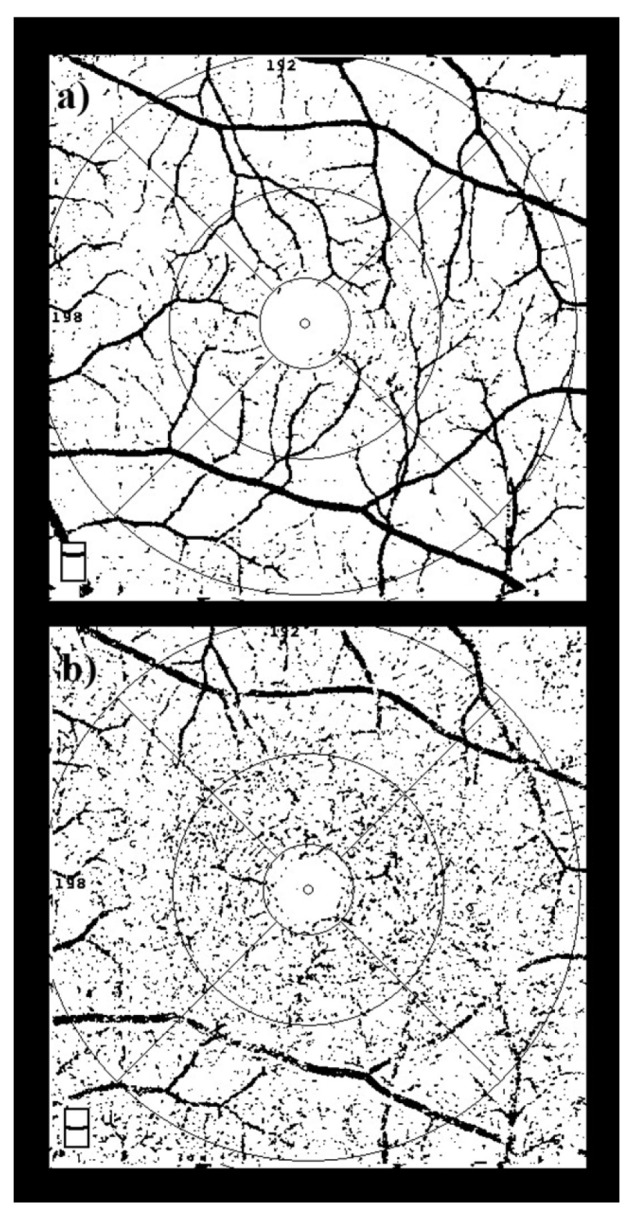
**Image Processing Steps for Fractal Dimension Analysis.** Workflow for calculating FD: OCTA images were exported, binarized, and skeletonized using ImageJ software. Subsequently, FD values were computed using Fractalyse software based on the box-counting algorithm. This procedure was applied separately to both superficial (**a**) and deep (**b**) capillary plexus slabs.

This value reflects the geometric complexity of the vascular network, where higher FD values represent greater branching complexity and integrity. Separate FD values were calculated for the superficial (FDsup) and deep (FDdeep) capillary plexuses, and they were subsequently used for statistical analysis [13]. FD quantification steps, including image binarization and skeletonization using ImageJ, are illustrated in Figure 2.

### 2.4. Drusen Size and Count Analysis

Drusen were identified and measured in a 6 × 6 mm en face OCTA slab. Image analysis was performed using ImageJ software (NIH, Bethesda, MD, USA). First, each image was converted to 8-bit grayscale. The drusen area was then segmented using the Auto Local Threshold function with the “Phansalkar” method, which is effective in enhancing low-contrast regions. This normalization process ensured consistent measurement scaling across all subjects. Total drusen area was calculated based on the binarized and thresholded images.

### 2.5. Statistical Analysis

All statistical analyses were performed using SPSS software (version 25.0; IBM Corp., Armonk, NY, USA). Descriptive statistics were reported as the mean ± standard deviation or median (interquartile range), as appropriate. Normality of continuous variables was assessed using the Kolmogorov–Smirnov test. Log-transformation was explored for all continuous variables to assess model assumptions. Among the analyzed parameters, only drusen area demonstrated a skewed distribution requiring log-transformation, while all other OCTA-derived and FD variables showed acceptable distributional properties and were therefore analyzed on their original scale. Group comparisons between eyes with large and small drusen were performed using independent-samples *t* tests or Mann–Whitney U tests, depending on data distribution. Associations between OCTA-derived parameters, FD indices, and drusen area were evaluated using Pearson or Spearman correlation coefficients, as appropriate. To account for inter-eye correlation in patients contributing both eyes, patient-level clustered analyses were performed. Linear mixed-effects models with random intercepts for patient identity were used to assess independent associations with log-transformed drusen area. Additionally, generalized estimating equation (GEE) logistic regression models were applied to evaluate predictors of large versus small drusen. All statistical tests were two sided, and a *p*-value < 0.05 was considered statistically significant.

## 3. Results

A total of 113 eyes from 73 patients with dry AMD were included in the analysis. Eyes were classified into large drusen (*n* = 56) and small drusen (*n* = 57) groups based on the median drusen area. There was no significant difference in age between the two groups (*p* = 0.98). As expected, the mean drusen area was significantly greater in the large drusen group compared with the small drusen group (3.10 ± 1.81 mm^2^ vs. 0.48 ± 0.33 mm^2^; *p* < 0.001). Global OCTA and FD parameters demonstrated that FDsup did not differ significantly between groups (*p* = 0.48). FDdeep showed a non-significant trend toward higher values in eyes with larger drusen (*p* = 0.07). In contrast, CCflow was significantly lower in the large drusen group compared with the small drusen group (*p* < 0.001) (Table 1).

No significant differences were observed between groups in superficial capillary plexus vessel density parameters across whole, parafoveal, perifoveal, or sectoral regions (all *p* > 0.05; Table 2). Similarly, deep capillary plexus vessel density parameters did not differ significantly between eyes with large and small drusen (all *p* > 0.05; Table 3). In univariable correlation analyses, neither FDsup nor FDdeep showed a significant correlation with drusen area (FDsup: ρ = 0.023, *p* = 0.809; FDdeep: ρ = 0.137, *p* = 0.148). In contrast, CCflow demonstrated a moderate and statistically significant negative correlation with drusen area (ρ = −0.452, *p* < 0.001) (Table 4).

In patient-level clustered multivariable analyses accounting for inter-eye correlation, CCflow remained independently associated with drusen burden. In the linear mixed-effects model with log-transformed drusen area as the outcome, lower CCflow was significantly associated with greater drusen burden (β = −0.759, *p* < 0.001), whereas FDdeep (*p* = 0.71) and age (*p* = 0.61) were not independently associated (Table 5). Consistently, in the generalized estimating equation logistic regression model, lower CCflow was associated with higher odds of large drusen (OR = 0.050, 95% CI 0.0085–0.288; *p* < 0.001). FDdeep (*p* = 0.613) and age (*p* = 0.869) were not significant predictors in the model (Table 6).

## 4. Discussion

In this study, we evaluated the relationship between OCTA-derived CCflow and retinal vascular fractal complexity with drusen burden in eyes with dry AMD without systemic comorbidities. Our principal finding is that reduced CCflow is robustly and independently associated with greater drusen burden after accounting for inter-eye correlation and age. In contrast, FD of the deep capillary plexus demonstrated a modest association with drusen area at the univariable level but did not remain independently associated in patient-level clustered multivariable analyses.

The inverse relationship between CCflow and drusen burden observed in this study is consistent with accumulating evidence implicating choriocapillaris dysfunction as a central component of dry AMD pathophysiology. Notably, the strong association between CCflow and drusen burden persisted despite manual correction of segmentation errors, supporting the robustness of this finding. In contrast, the weaker correlations observed for FD metrics may partly reflect their greater sensitivity to local microstructural variability and qualitative image-dependent factors. Several OCTA-based studies have demonstrated that CCflow deficits precede the development of geographic atrophy and correlate with functional impairment in nonexudative AMD [20,23,24,25]. Our findings extend this body of literature by demonstrating that reduced CCflow is strongly associated with drusen burden even in the absence of overt atrophy or neovascularization, supporting the concept that impaired choroidal perfusion contributes to early metabolic stress and drusen accumulation.

FD analysis revealed a weak, non-significant positive trend between deep capillary plexus complexity and drusen area. This finding may reflect localized microvascular remodeling in response to outer retinal stress or altered metabolic demand associated with subretinal pigment epithelium deposits. However, the absence of an independent association after adjustment for age and inter-eye correlation in our clustered models indicates that fractal complexity should be interpreted as a secondary descriptive parameter rather than a stand-alone biomarker of disease severity.

Superficial capillary plexus FD was not correlated with drusen size; this supports previous observations that the superficial retinal vascular structure remains relatively preserved, including in more advanced or exudative forms of AMD [26]. This layer-specific vulnerability underscores the value of stratified OCTA analysis and suggests that deeper retinal and choroidal compartments may be more sensitive to early pathological changes in AMD.

The positive correlation between FDdeep and drusen size suggests that deep capillary plexus complexity may increase in response to outer retinal stress or ischemia induced by sub-RPE deposits. This observation aligns with previous studies using OCTA and fractal analysis, which have described increased vascular branching complexity as a potential early adaptive response in AMD before the onset of geographic atrophy. For instance, Young et al. [27] demonstrated that eyes with intermediate AMD showed elevated FD values, particularly in the deep capillary plexus, compared with healthy controls, hypothesizing that this may reflect microvascular remodeling. Zahid et al. [13] further demonstrated the sensitivity of FD metrics to microvascular alterations in diabetic and ischemic retinal diseases. In a study by Talu et al. [28] in patients with diabetic macular edema, multifractal geometry and lacunarity analyses supported the broader application of fractal-based measures for characterizing microvascular disorganization. In this context, our findings suggest that FDdeep may provide complementary descriptive information regarding vascular complexity in dry AMD rather than serving as an independent biomarker.

In the context of neovascular AMD, Al-Sheikh et al. [29] demonstrated that FD values were significantly reduced in inactive and post-treatment lesions compared with active neovascular membranes, highlighting the sensitivity of fractal metrics to vascular remodeling and disease activity. In contrast, Serra et al. [30] reported that FD alone did not reliably differentiate between complex neovascular entities such as polypoidal choroidal neovascularization and type 1 choroidal neovascularization, underscoring its limitations in specific heterogeneous vascular scenarios.

From a clinical perspective, CCflow and FD metrics appear to provide complementary information. While conventional OCTA parameters such as vessel density and foveal avascular zone measurements are widely used, they may be less sensitive to early microvascular dysfunction [31]. CCflow offers a direct estimate of choriocapillaris perfusion integrity, whereas fractal analysis captures geometric aspects of vascular organization that may reflect adaptive remodeling. Significantly, by excluding patients with systemic vascular comorbidities such as hypertension and diabetes, our study minimizes confounding effects known to influence OCTA measurements, allowing a more AMD-specific assessment of microvascular alterations.

This study has several strengths, including a carefully selected cohort of systemically healthy eyes with dry AMD, standardized image processing and quantification methods, and the use of patient-level clustered statistical models to account for inter-eye correlation. Nevertheless, limitations should be acknowledged. The cross-sectional design precludes causal inference, and longitudinal studies are needed to determine whether changes in CCflow or fractal metrics predict progression to geographic atrophy or advanced AMD. In addition, the absence of standardized normative ranges and clinical cutoff values for CCflow and FD limits their immediate application in routine clinical practice. Methodological variability among OCTA devices and image processing pipelines may also affect inter-study comparability. Given the cross-sectional design of the present study, causal or temporal relationships cannot be inferred, and longitudinal studies are required to determine whether reductions in CCflow precede drusen accumulation or represent downstream pathological changes.

Another crucial methodological strength of the present study is the deliberate inclusion of a systemically healthy cohort of elderly patients with dry AMD, in whom major systemic confounders such as hypertension and diabetes mellitus were strictly excluded. This approach is particularly relevant given that most patients with AMD are older and frequently harbor systemic comorbidities that independently affect retinal and choroidal microvasculature. In much of the existing OCTA literature, these confounding conditions are either incompletely controlled or only statistically adjusted for, rather than being eliminated at the cohort selection level. Consequently, reported associations between vascular metrics and AMD-related features may partially reflect systemic vascular disease rather than AMD-specific microvascular remodeling.

In this context, the absence of an independent association between deep capillary plexus FD and drusen burden in our clustered multivariable analyses may be attributable to the relative vascular homogeneity of our carefully selected population. FD metrics are known to be sensitive to generalized microvascular alterations and may therefore appear more prominently in cohorts where systemic vascular disease is prevalent [32]. By minimizing such confounding influences, our study isolates microvascular changes more directly attributable to AMD itself, which may explain why fractal complexity did not emerge as an independent biomarker in this setting. These findings underscore the importance of rigorous cohort definition when interpreting OCTA-based biomarkers and suggest that some previously reported associations in the literature may be driven, at least in part, by unaccounted systemic vascular confounders rather than disease-specific mechanisms.

## 5. Conclusions

In conclusion, reduced CCflow is independently associated with greater drusen burden in eyes with dry AMD, supporting its role as a robust quantitative indicator of choroidal compromise. Deep capillary plexus fractal complexity shows a modest univariable association with drusen burden. Still, it does not remain independently associated after adjustment for age and inter-eye correlation, suggesting a complementary descriptive role. Together, these OCTA-derived parameters provide distinct yet complementary insights into early retinal choroidal remodeling in dry AMD and may enhance future phenotyping and monitoring strategies when integrated into multimodal imaging frameworks.

## Figures and Tables

**Table 1:** Comparison of Global OCTA and Fractal Parameters Between Small and Large Drusen Groups.

Parameter	Large Drusen (Mean ± SD)	Small Drusen (Mean ± SD)	*p*-Value (Unadjusted)
Superficial Fractal Dimension (FDsup)	1.52 ± 0.04	1.51 ± 0.05	0.48
Deep Fractal Dimension (FDdeep)	1.54 ± 0.08	1.51 ± 0.09	0.07
Outer retina Flow Area (ORflow)	23.54 ± 171.23	0.56 ± 0.39	0.18
Choriocapillaris Flow Area (CCflow)	1.71 ± 0.26	1.92 ± 0.18	<0.001 *
Foveal avascular zone (FAZ)	0.29 ± 0.13	0.30 ± 0.10	0.55
FAZ perimeter	2.08 ± 0.49	2.17 ± 0.38	0.28
Foveal density	51.54 ± 4.62	51.57 ± 4.83	0.97

*, statistically significant. Group comparisons are descriptive and unadjusted for inter-eye correlation.

**Table 2:** Superficial Layer Vessel Density Parameters Between Small and Large Drusen Groups.

Layer	Parameter	Large Drusen (Mean ± SD)	Small Drusen (Mean ± SD)	*p*-Value
Superficial	Whole density	47.98 ± 3.54	48.54 ± 3.57	0.53
Superficial	Superior hemisphere	47.80 ± 3.50	48.20 ± 3.69	0.77
Superficial	Inferior hemisphere	48.15 ± 3.79	48.88 ± 3.63	0.43
Superficial	Fovea	19.14 ± 6.50	18.23 ± 7.01	0.19
Superficial	Parafovea	50.04 ± 3.95	51.03 ± 3.88	0.23
Superficial	Parafoveal superior hemisphere	50.05 ± 3.98	50.95 ± 4.21	0.26
Superficial	Parafoveal inferior hemisphere	50.03 ± 4.40	51.13 ± 3.89	0.19
Superficial	Parafoveal temporal	50.28 ± 3.87	51.22 ± 3.75	0.27
Superficial	Parafoveal superior	50.50 ± 4.69	51.58 ± 4.67	0.27
Superficial	Parafoveal nasal	49.08 ± 4.94	49.67 ± 4.44	0.67
Superficial	Parafoveal inferior	50.16 ± 5.08	51.12 ± 7.07	0.09
Superficial	Perifovea	48.57 ± 3.60	49.56 ± 4.29	0.40
Superficial	Perifoveal superior hemisphere	48.45 ± 3.38	49.05 ± 3.84	0.68
Superficial	Perifoveal inferior hemisphere	48.69 ± 4.02	49.53 ± 3.96	0.34
Superficial	Perifoveal temporal	45.05 ± 3.81	45.91 ± 3.89	0.36
Superficial	Perifoveal superior	48.20 ± 3.38	48.65 ± 4.17	0.85
Superficial	Perifoveal nasal	52.45 ± 3.86	53.32 ± 3.55	0.34
Superficial	Perifoveal inferior	48.71 ± 4.30	49.35 ± 4.80	0.31

**Table 3:** Deep Layer Vessel Density Parameters Between Small and Large Drusen Groups.

Layer	Parameter	Large Drusen (Mean ± SD)	Small Drusen (Mean ± SD)	*p*-Value
Deep	Whole density	48.96 ± 5.52	48.61 ± 6.00	0.61
Deep	Inferior hemisphere	49.32 ± 5.77	49.06 ± 5.94	0.73
Deep	fovea	34.34 ± 7.23	33.41 ± 6.70	0.37
Deep	Parafovea	52.62 ± 5.26	53.07 ± 4.14	0.76
Deep	Parafoveal superior hemisphere	52.92 ± 5.24	53.27 ± 4.37	0.83
Deep	Parafoveal inferior hemisphere	52.32 ± 5.46	52.89 ± 4.16	0.69
Deep	Parafoveal temporal	53.68 ± 5.09	53.56 ± 6.06	0.91
Deep	Parafoveal superior	52.14 ± 6.14	52.58 ± 5.08	0.87
Deep	Parafoveal nasal	53.33 ± 4.92	63.31 ± 72.32	0.66
Deep	Parafoveal inferior	51.35 ± 6.18	51.78 ± 4.80	0.86
Deep	Perifovea	50.16 ± 6.06	49.78 ± 6.68	0.67
Deep	Perifoveal superior hemisphere	49.84 ± 6.06	49.54 ± 6.90	0.79
Deep	Perifoveal inferior hemisphere	50.46 ± 6.35	50.02 ± 6.78	0.60
Deep	Perifoveal temporal	53.28 ± 5.41	52.75 ± 5.62	0.52
Deep	Perifoveal superior	48.73 ± 6.56	48.58 ± 8.01	0.98
Deep	Perifoveal nasal	48.68 ± 6.49	48.12 ± 6.78	0.65
Deep	Perifoveal inferior	49.85 ± 7.26	49.60 ± 7.74	0.87

**Table 4:** Correlation between Drusen Area and FD/CCflow Parameters.

Parameter	ρ (rho)	*p*-Value
Superficial Fractal Dimension (FDsup)	0.023	0.809
Deep Fractal Dimension (FDdeep)	0.137	0.148
Choriocapillaris Flow Area (CCflow)	−0.452	<0.001 *

Spearman correlation analysis. Statistically significant at *p* < 0.05.*, statistically significant.

**Table 5:** Linear Mixed-Effects Model.

Predictor	β	95% CI	*p*-Value
CCflow	−0.759	[−1.16, −0.36]	<0.001 *
FDdeep	0.19	[−0.80, 1.18]	0.71
Age	0.004	[−0.01, 0.02]	0.61

*, statistically significant.

**Table 6:** GEE Logistic Regression.

Predictor	OR	95% CI	*p*-Value
CCflow	0.050	[0.0085–0.288]	<0.001 *
FDdeep	2.36	[0.085–65.39]	0.613
Age	0.996	[0.946–1.048]	0.869

Outcome: Large vs. Small Drusen; *, statistically significant. Generalized estimating equation (GEE) logistic regression with exchangeable working correlation and robust standard errors, accounting for inter-eye correlation. Odds ratios represent the effect per unit increase in continuous predictors.

## Data Availability

The data presented in this study are available on request from the corresponding author.
